# Supplementary material for: PLK-1 Interacting Checkpoint Helicase, PICH, Mediates Cellular Oxidative Stress Response
Source: Epigenomes. 2022 Oct 18;6(4):36. doi: 10.3390/epigenomes6040036 (PMC9590091; doi:10.3390/epigenomes6040036)
Supplement: Supplementary file 1 [file epigenomes-06-00036-s001.zip › epigenomes-1766544-Supplementary.pdf]

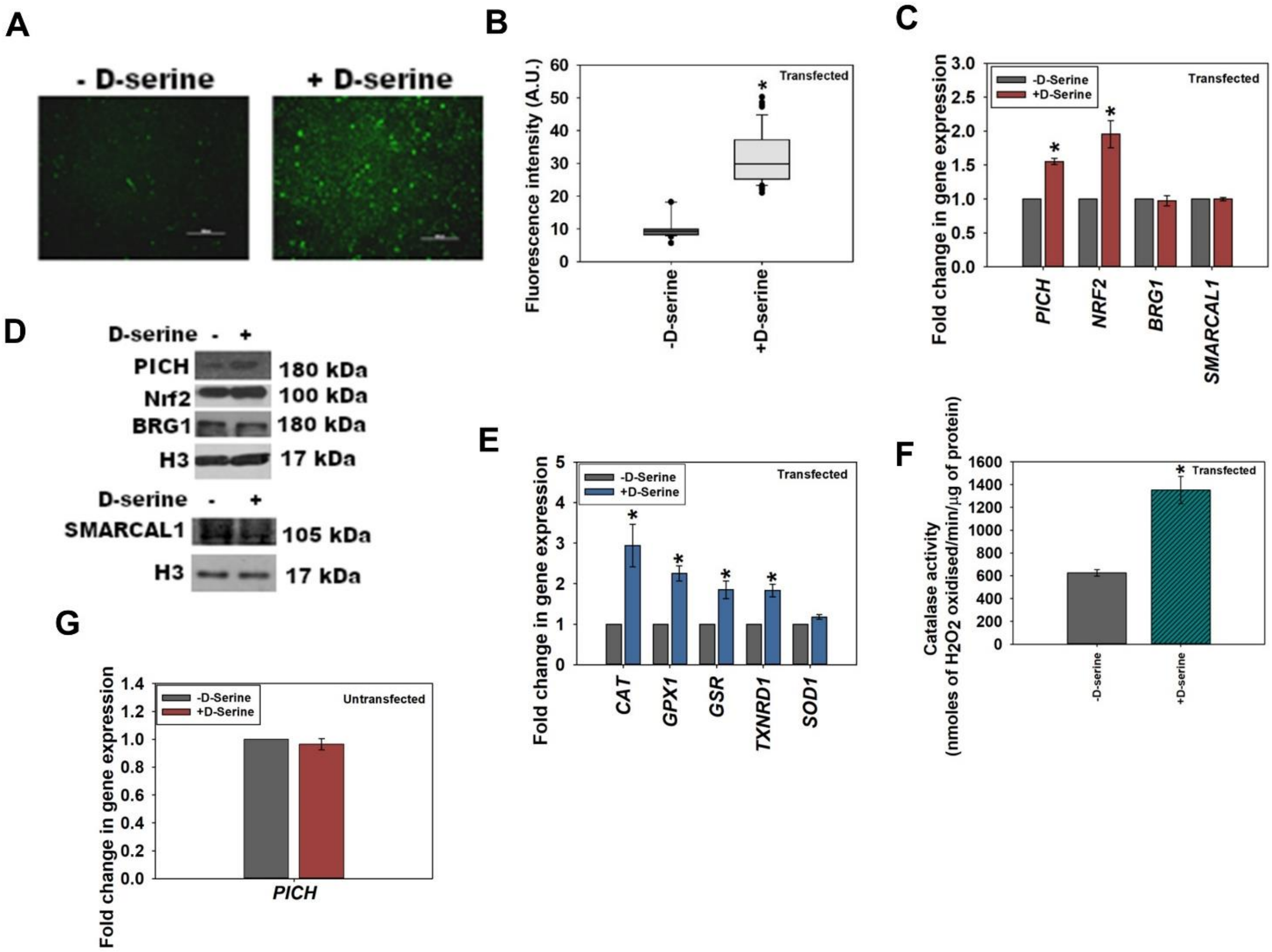

Supplementary Figure S1. *PICH* expression is upregulated when cells are exposed to oxidative stress. (A). Cellular ROS levels were measured using DCFDA in *DAAO* transfected HeLa cells in the absence and presence of D-serine. (B). Quantitation of the fluorescence intensity. (C). Expression of *PICH*, *Nrf2*, *BRG1*, and *SMARCAL1* was analyzed by qRT-PCR. (D). Expression of PICH, Nrf2, BRG1, and SMARCAL1 was analyzed by westernblot using H3 as control. (E). Expression of *CAT, GPX1, GSR, TXNRD1,* and *SOD1* was analyzed by qRT-PCR. (F). Catalase activity (µmol/min) was examined in the absence and presence of D-serine treatment in DAAO transfected HeLa cells. (G). Expression of *PICH* was analyzed by qRT-PCR in untransfected HeLa cells after treatment with 25mM D-Serine for 10 min. .

In these experiments, *DAAO* transfected cells were treated with 25 mM D-serine for 10 min to induce ROS production. *GAPDH* was used as the internal control in the qRT-PCR experiments. The qRT-PCR experiments are presented as average ± SEM of three independent experiments. The western blots are presented as average ± SEM of three independent experiments. The catalase activity is presented as average ± SEM of three independent experiments.

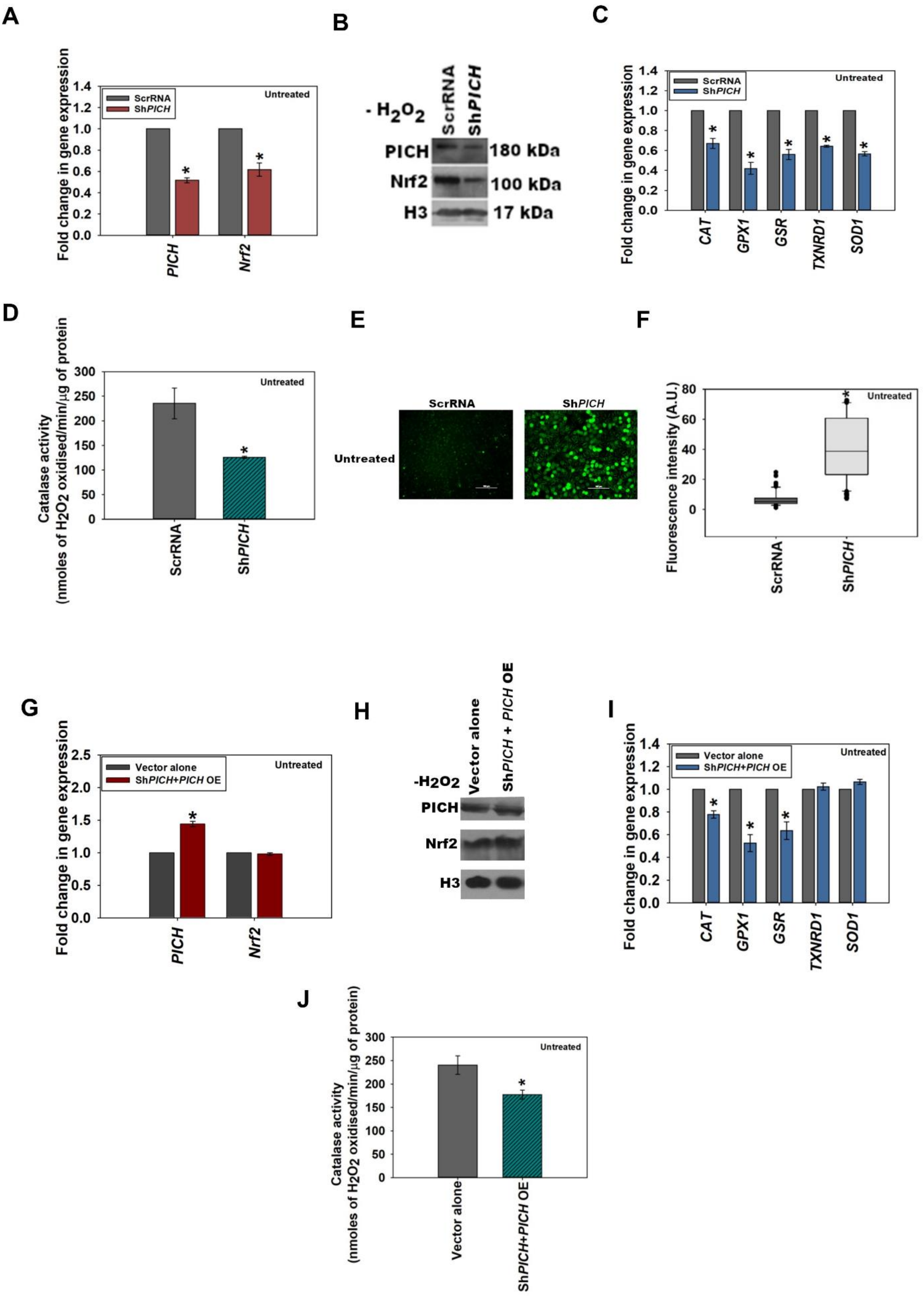
A
Fold change in gene expression
ScrRNA
ShPICH
Untreated
PICH
Nrf2
B
- H2O2
ScrRNA
ShPICH
PICH
180 kDa
Nrf2
100 kDa
H3
17 kDa
C
Fold change in gene expression
ScrRNA
ShPICH
Untreated
CAT
GPX1
GSR
TXNRD1
SOD1
D
Catalase activity
(nmoles of H2O2 oxidised/min/μg of protein
Untreated
ScrRNA
ShPICH
E
ScrRNA
ShPICH
Untreated
F
Fluorescence intensity (A.U.)
Untreated
ScrRNA
ShPICH
G
Fold change in gene expression
Vector alone
ShPICH+PICH OE
Untreated
PICH
Nrf2
H
-H2O2
Vector alone
ShPICH + PICH OE
PICH
Nrf2
H3
I
Fold change in gene expression
Vector alone
ShPICH+PICH OE
Untreated
CAT
GPX1
GSR
TXNRD1
SOD1
J
Catalase activity
(nmoles of H2O2 oxidised/min/μg of protein)
Untreated
Vector alone
ShPICH+PICH OE

Supplementary Figure S2. PICH regulates the expression of *Nrf2* in HeLa cells in the absence and presence of oxidative stress. (A). Expression of *PICH* and *Nrf2* in HeLa cellstransfected with either ScrRNA or with Sh*PICH* plasmid in HeLa cells in the absence of H2O2 treatment (P-value = 3E-10 for Sh*PICH*). (B). Expression of PICH and Nrf2 in untreated HeLa cells transfected with either ScrRNA or with Sh*PICH* was analyzed by western blot. (C). Expression of antioxidant genes *CAT, GPX1, GSR, TXNRD1,* and *SOD1* were quantitated by qRT-PCR in untreated HeLa cells transfected either with ScrRNA or with Sh*PICH.* (D). Catalase activity (μmol/min) was quantitated in untreated HeLa cells transfected either with ScrRNA or with Sh*PICH.* (E). Cellular ROS was analyzed using DCFDA in HeLa cells transfected with either ScrRNA or with Sh*PICH* plasmid. (F). Fluorescent intensity was quantitated using the software provided by TiE, Nikon Microscope.(G). Transcript levels of *PICH* and *Nrf2* were quantitated by qRT-PCR in untreated HeLa cells transfected with ScrRNA and empty vector or with the Sh*PICH* and *PICH* overexpression construct. (H). Expression of PICH and Nrf2 in untreated HeLa cells transfected with ScrRNA and empty vector or with the Sh*PICH* and *PICH* overexpression construct was analyzed by western blot. (I). Expression of antioxidant genes *CAT, GPX1, GSR, TXNRD1,* and *SOD1,* were quantitated in untreated HeLa cells transfected with ScrRNA and empty vector or with the Sh*PICH* and *PICH* overexpression construct. (J). Catalase activity (μmol/min) was estimated in untreated HeLa cells transfected with ScrRNA and empty vector or with the Sh*PICH* and *PICH* overexpression construct.

GAPDH was used as the internal control in the qRT-PCR experiments. The qRT-PCR experimentsare presented as average ± SEM of three independent experiments. The western blots are presented as average ± SEM of three independent experiments. The catalase activity is presented as average ± SEM of three independent experiments.

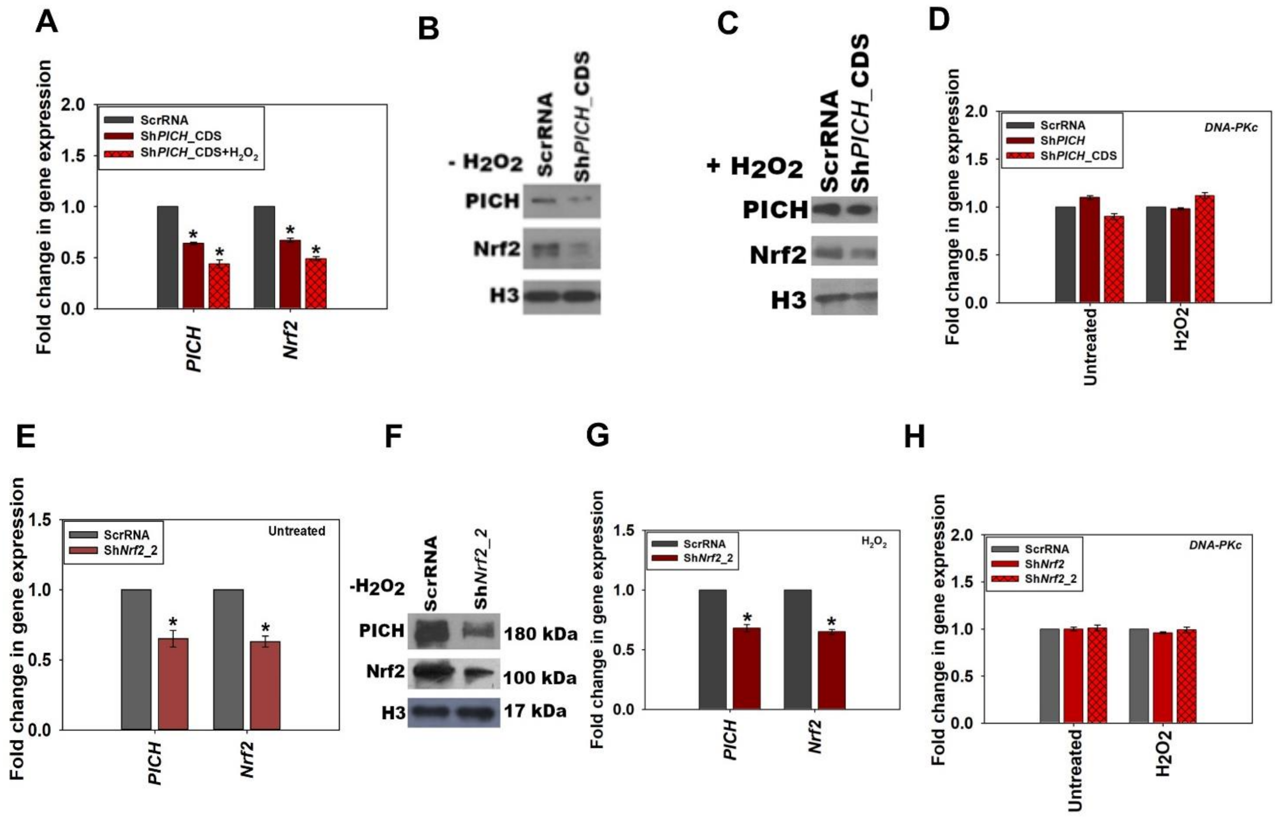

Supplementary Figure S3. ***ShPICH_2*** **regulates the expression of** ***Nrf2*** **in HeLa cells in the absence and presence of oxidative stress.** (A). Expression of *PICH* and *Nrf2* in HeLa cells transfected with either ScrRNA or with Sh*PICH_CDS* plasmid in HeLa cells in the absence and in presence of of H2O2 treatment. (B). Expression of PICH and Nrf2 in untreated HeLa cells transfected with either ScrRNA or with Sh*PICH*_CDS was analyzed by western blot. (C). Expression of PICH and Nrf2 in treated HeLa cells transfected with eitherScrRNA or with Sh*PICH*_CDS was analyzed by western blot (D). Expression of *DNA-PKc* in untreated and in treated (100 M H2O2; 20 min) HeLa cells transfected either with ScrRNA or with Sh*PICH and* Sh*PICH_CDS* construct. (E). Expression of *PICH*, *Nrf2* in untreated HeLa cells transfected either with ScrRNA or with Sh*Nrf2_2* construct. (F). Expression of PICH and Nrf2 in untreated HeLa cells transfected with either ScrRNA or with Sh*Nrf2_2* was analyzed by western blot. (G). Expression of *PICH*, *Nrf2* in treated (100 M H2O2; 20 min) transfected either with ScrRNA or with Sh*Nrf2_2*. (H). Expression of *DNA-PKc* in untreated and in treated (100 M H2O2; 20 min) HeLa cells transfected either with ScrRNA or with Sh*Nrf2 and* Sh*Nrf2_2* construct.

GAPDH was used as the internal control in the qRT-PCR experiments. The qRT-PCR experiments are presented as average ± SEM of three independent experiments. The western blots are presented as average ± SEM of two independent experiments.

Conditions/Genes	*PICH*	*Nrf2*
H_2O_2 treated (T)	1.65	4.4
Sh*PICH*	0.51	0.61
Sh*PICH*+ T	0.74	0.66
Sh*PICH*+ *PICH* OE	1.44	0.98
(Sh*PICH* + *PICH* OE)+T	1.2	1.41
Sh*PICH*+ *PICHK128A*	0.48	0.6
(Sh*PICH*+ *PICHK128A*)+ T	0.58	0.67

U Untreated
T Treated

<0.5 <0.65 <0.8 ~1 >1.5

Conditions/Genes	*CAT*	*GPX1*	*GSR*	*TXNRD1*	*SOD1*
H_2O_2 treated (T)	2.9	10	1.12	5.7	6.2
Sh*PICH*	0.67	0.42	0.56	0.64	0.56
Sh*PICH*+ T	0.71	0.46	0.72	0.58	0.71
Sh*PICH*+ *PICH* OE	0.77	0.52	0.63	1.02	1.06
(Sh*PICH* + *PICH* OE)+T	1.03	1.09	0.96	1.01	0.99
Sh*PICH*+ *PICHK128A*	0.64	0.53	0.74	0.66	0.6
(Sh*PICH*+ *PICHK128A*)+ T	0.69	0.6	0.76	0.61	0.69

U Untreated
T Treated

<0.5 <0.6 <.65 <0.75 <0.8 ~1 >2.5 >5 >6 10

Supplementary Figure S4. Heat maps of the fold changes found in qRT-PCR analysis.

A

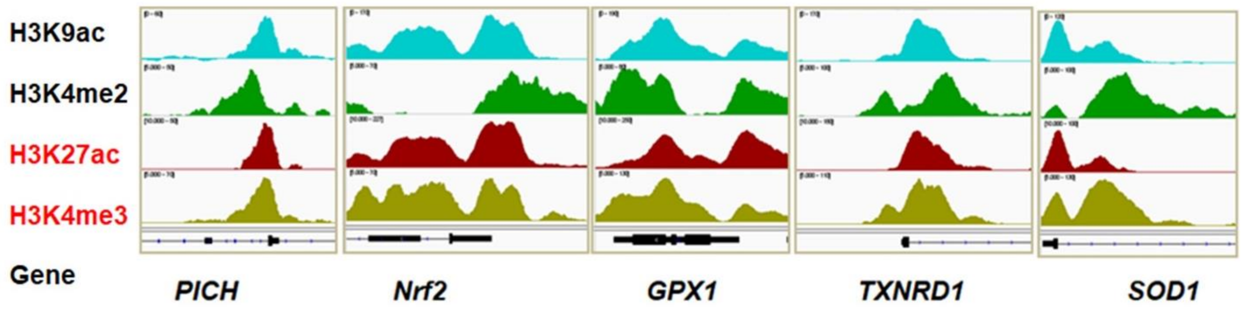

B

Promoters	PICH U	PICH T	Nrf2 U	Nrf2 T	RNAPII U	RNAPII T	H3K4me3 U	H3K4me3 T	H3K27ac U	H3K27ac T
PICH	0.92	1.65	0.5	2.11	1.23	1.74	0.72	7.57	18.9	47.1
Nrf2	7.79	2.8	8.06	0.9	0.47	2.63	0.95	0.85	0.95	2.72
GPX1	0.54	6.07	3.14	8.74	0.9	2.39	1.29	3.56	0.83	1.71
TXNRD1	1.58	3.25	3.62	11.54	3.49	8.39	2.67	30.2	2.58	6.15
SOD1	1.73	3.01	1.32	6.92	0.09	4.18	1.35	5.17	1.12	3.18

U Untreated
T Treated

Decrease in binding
Increase in binding
Basal level of binding

Supplementary Figure S5. Histone marks associated with transcription activation are enriched on PICH, Nrf2, and antioxidant gene promoters on oxidative stress. (A). ChIP-seq peaks of histone modification marks (H3K4me1, H3K4me3, H3K27ac, and H3K9c) werevisualized using IGV track on the promoters of *PICH*, *Nrf2*, *GPX1*, *TXNRD1*, and *SOD1*. (B). Heat map of the fold enrichment values found in the ChIP experiments.

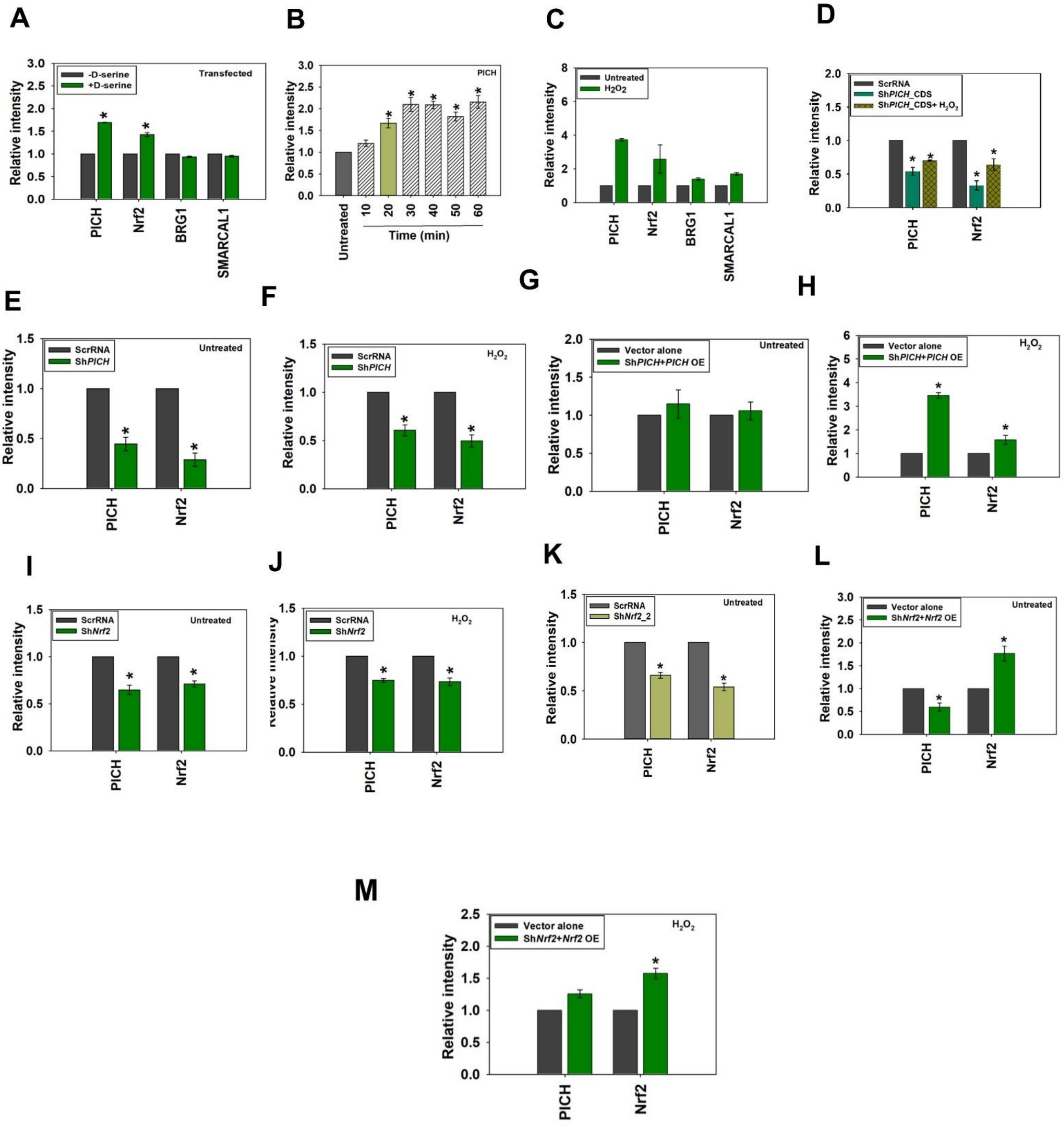

Supplementary Figure S6. Quantification of all western blots. The western blots are presented as average ± SEM of two independent experiments. The intensities were quantitated using Image J software. The intensities of the proteins were normalized with respect to H3 and are represented as Relative Intensity on the y-axis.

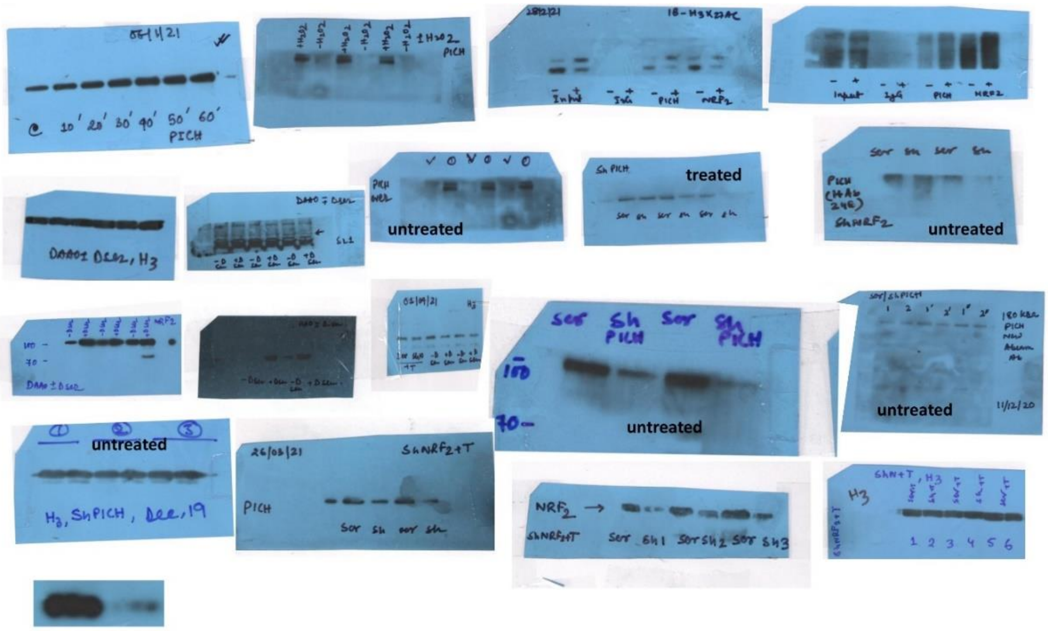

NRF2,ShPICH+T

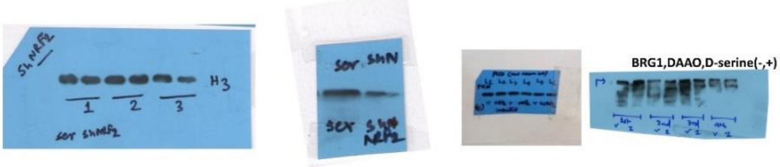

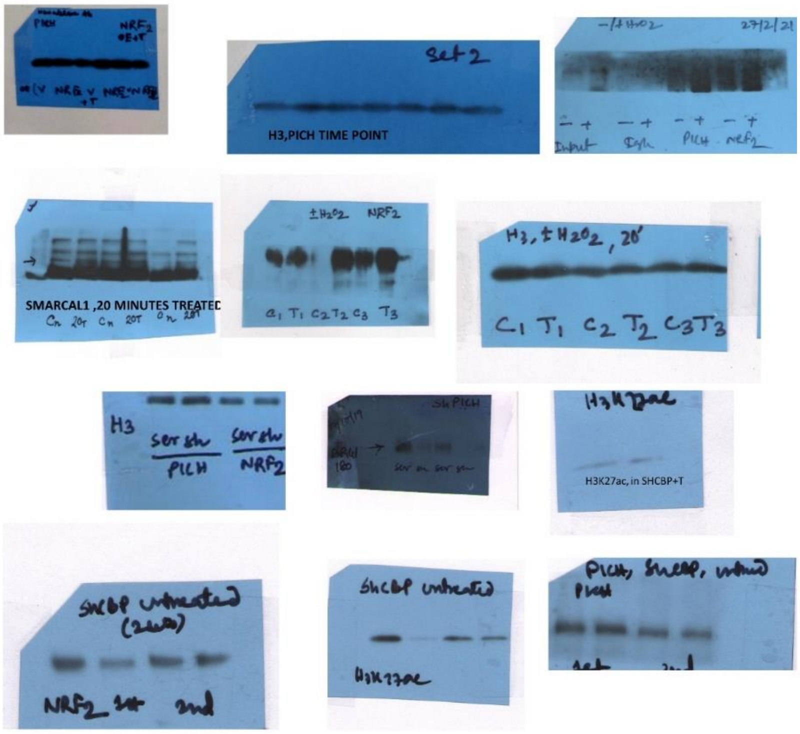

Nrf2,in Sh PICH_2

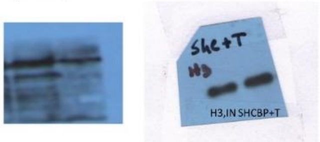

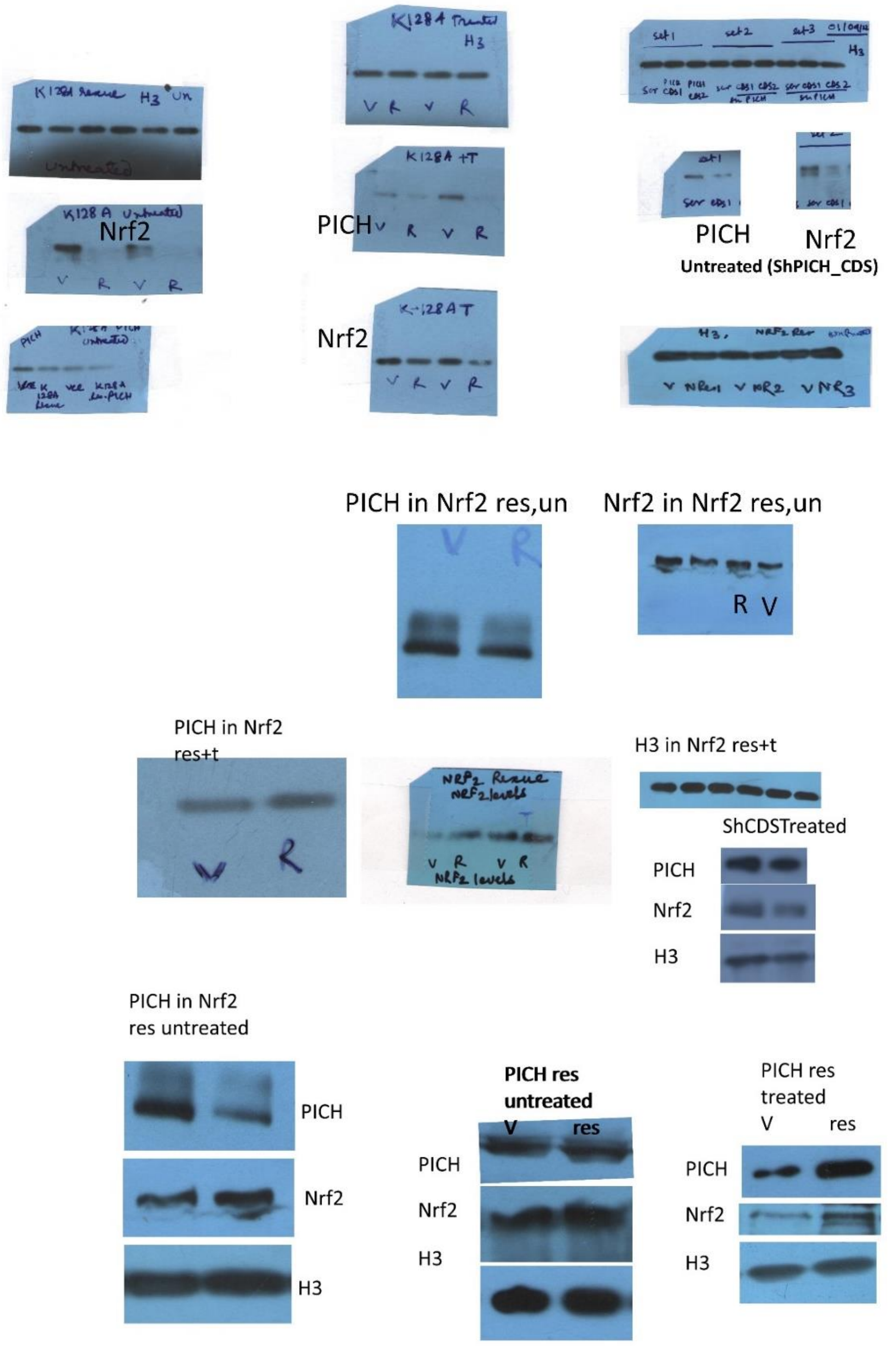

Supplementary Figure S7. Original western blots.

Supplementary Table S1: List of primers used in qRT-PCR experiments.

Gene name	Forward primer (5' 3')	Reverse primer (5' 3')
PICH	GGTAACTGGAACCCAATCCGAGG	TCTTTCACATATCTTAGGTAATG
NRF2	GCGACGGAAAGAGTATGAGC	GTTGGCAGATCCACTGGTTT
BRG1	CGTGGAGGAGAAGAAGAAGA	CTGTTTGAGGACACCATTGA
SMARCAL1	GAAGTGGAGCTTTCTCTTGG	CAGATTAGACACGAGCTTGG
CAT	AGCTTAGCGTTCATCCGTGT	TCCAATCATCCGTCAAAACA
GPX1	CTCTTCGAGAAGTGCGAGGT	TCGATGTCAATGGTCTGGAA
GSR	AGTGGGACTCACGGAAGATG	TTCACTGCAACAGCAAAACC
TXNRD1	CTAAAAATGAACGGCCCTGA	ACACATGTTCCTCCGAGACC
SOD1	AGGGCATCATCAATTTCGAG	ACATTGCCCAAGTCTCCAAC
GAPDH	GGTCGGAGTCAACGGATTTGGTC	GAGGGATCTCGCTCCTGGAAG
DNA-PKc	GCCAGAGAAGCAGCAAATGG	TCTCCGAAAACGACCAGTGG

Supplementary Table S2: List of primers used in ChIP experiments.

Gene name	Forward primer (5' 3')	Reverse primer (5' 3')
PICH Pair 1	AGCGGTCCAGACATACCTTA	GAGCGAAATTCAAGCTCCAAAC
PICH Pair 2	GTGATCTCTGCCTTCGAGAC	CCGCAATGACTGGGATCATA
*Nrf*2 Pair 1	ACTCGGTAATCGGCTACA	CGAGCTTCTTGCGTCAG
*Nrf*2 Pair2	GCTGACGCAAGAAGCTC	CCTTCGCAAACAACTCTTTATC
*GPX*1 Pair 1	AAACTGGTTGCACGGGAAG	TGTGTGCTGCTCGGCTA
*GPX*1 Pair 2	CATGGCGCAATTGTCCAAGAA	TCCTTCCGGCTTAGGAGGAG
TXNRD1 Pair 1	GCACGAGGAGTGGATTTC	TGAGAATGATGAAGACATCAGG
TXNRD1 Pair 2	GAGTTCTGTAGCTACTGCCTTA	AAAGCAGAAATCCACTCCTC
SOD1 Pair 1	GGGTTCTGGACGTTTCC	CGACTACTTTATAGGCCAGAC
SOD1 Pair 2	CGGAGGTCTGGCCTATAA	CTTCTGCTCGAAATTGATGATG
DNA-PKc Pair 1	AACTCTTGACCTAGGCCCCT	CAGTAAGCGCGCCTCTTTG
GAPDH Pair 1	AAAAGCGGGGAGAAAGTAGG	AAAAGCGGGGAGAAAGTAGG

Supplementary Table S3: Oligonucleotides used in PICH-DNA interaction studies. The ARE sequence is highlighted in Red color. Boldand underlined residue shows the changes made with respect to GPX1 ARE.

**Oligonucleotide**	**Forward sequence** (5' 3')	**Reverse sequence** (5' 3')	**Mfold** (G kcal/mol)	**G-Quadruplex(G-score)**
**Stem-loop DNA (slDNA)**	GCGCAATTGCGCTCGACGATTTT TTAGCGCAATTGCGC		-16.3	0
dsDNA	GCGCAATTGCGC	CGCGTTAACGCG	-4.15	0
PICH	ACCCAATCCGAGGGTCATGGAGG CATCCCGAAGGTTTC	GAAACCTTCGGGATGCCTCCATGACCCTC GGATTGGGT	-3.18	13
Nrf2	GACCGCGAGCTTCTTGCGTCAGC CCCGGCGCGGGTGGG	CCCACCCGCGCCGGGGCTGACGCAAGAA GCTCGCGGTC	-6.41	0
SOD1	CCAGGACCTCGGCGTGGCCTAGC GAGTTATGGCGACGA	TCGTCGCCATAACTCGCTAGGCCACGCCG AGGTCCTGG	-5.50	11
GPX1	TGTGGCGTCCCTCTGAGGCACCA CGGTCCGGGACTACA	TGTAGTCCCGGACCGTGGTGCCTCAGAGG GACGCCACA	-2.88	14
TXNRD1	TTCTCGTAGCCATTAGGAAACAG CAACCCTTTCACCTC	GAGGTGAAAGGGTTGCTGTTTCCTAATGG CTACGAGAA	-0.98	0

Supplementary Table S4: List of target sequences used in making ShRNA constructs.

**Constructs**	**Target sequences** (5' 3')
Sh*PICH*	TATTCTGAGCACTAGCTTAAT
Sh*Nrf2*	GCTCCTACTGTGATGTGAAAT
Sh*Nrf2_2*	GGAGTGTCAGTATGTTGAATC
Sh*PICH*_CDS	GCTGCTCATTACCTAAGATAT
